# Supplementary figures and images for: Stress/Immune Biomarkers in Saliva among Children with ADHD Status
Source: Int J Environ Res Public Health. 2021 Jan 18;18(2):769. doi: 10.3390/ijerph18020769 (PMC7831062; doi:10.3390/ijerph18020769)

Figure S1. Flow chart of the study.

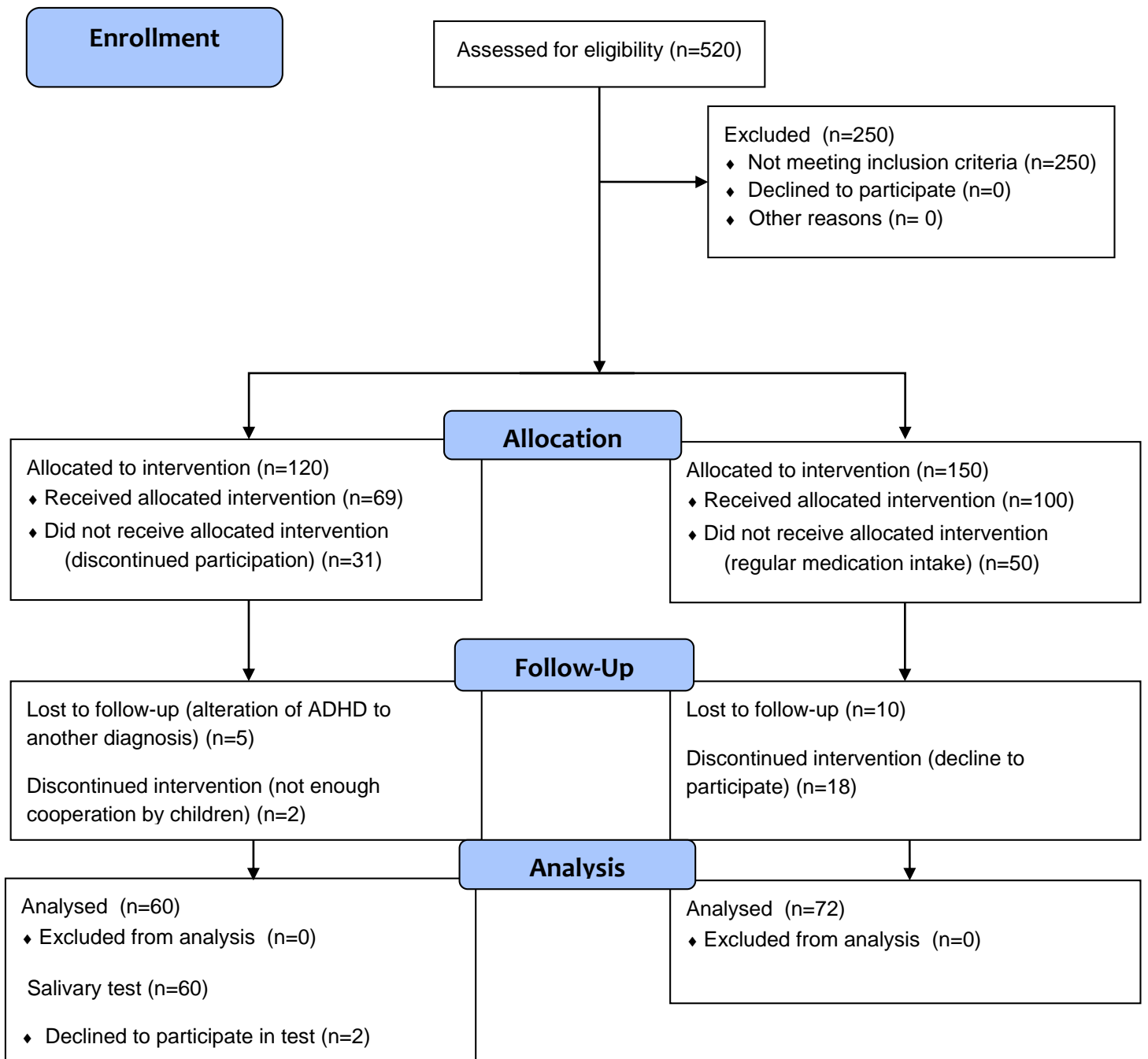

Supplement: Supplementary file 1 [file ijerph-18-00769-s001.pdf]
